# Regioselective ester cleavage during the preparation of bisphosphonate methacrylate monomers

**DOI:** 10.3762/bjoc.7.46

**Published:** 2011-03-25

**Authors:** Kamel Chougrani, Gilles Niel, Bernard Boutevin, Ghislain David

**Affiliations:** 1 Institut Charles Gerhardt, UMR 5253 CNRS, Ecole Nationale Supérieure de Chimie, 8, rue de l'Ecole Normale, 34296 Montpellier Cedex 5, France

**Keywords:** bifunctional monomer, phosphonic acid, regioselective ester cleavage

## Abstract

New functional monomers bearing a methacrylate, a bisphosphonate function and, for most, an internal carboxylate group, were prepared for incorporation into copolymers with adhesive or anticorrosive properties. Methanolysis of some trimethylsilyl bisphosphonate esters not only deprotects the desired bisphosphonate function but also regioselectively cleaves the alkyl ester function without affecting the methacrylate ester.

## Introduction

The potential applications for polymer products containing phosphorus are numerous; dental adhesives, ion-exchange resins and adhesion promotors are just three of the more common applications [1–7]. Compounds containing phosphorous are excellent promotors with respect to adhesion, and thus anti-corrosion. Commercial anti-corrosion polymer compounds are generally formed from Sipomer^®^ or Phosmer^®^ monomers, which are phosphate-type (meth)acrylates, and can be readily polymerized by emulsion or solution methods [8–9]. Polymers with some phosphonate functionality have long been established as excellent adhesives and anti-corrosion compounds [10–17], however, there has been very little investigation into the use of *phosphonate*-type methacrylates for the same purpose [8–9]. In the domain of polymer-based materials exhibiting specific properties, bifunctional monomers bearing a methacrylate function and a bisphosphonate function are recognized as useful building blocks for dental materials [11–12,18–19]. Such materials require a high hydrolytical stability that originates in the hydrolytical stability of the monomers. With this requirement in mind, we have investigated the synthesis of bisphosphonates and their deprotection to the corresponding acids.

## Results and Discussion

### Synthesis of bisphosphonate methacrylate monomers

**Scheme 1:**
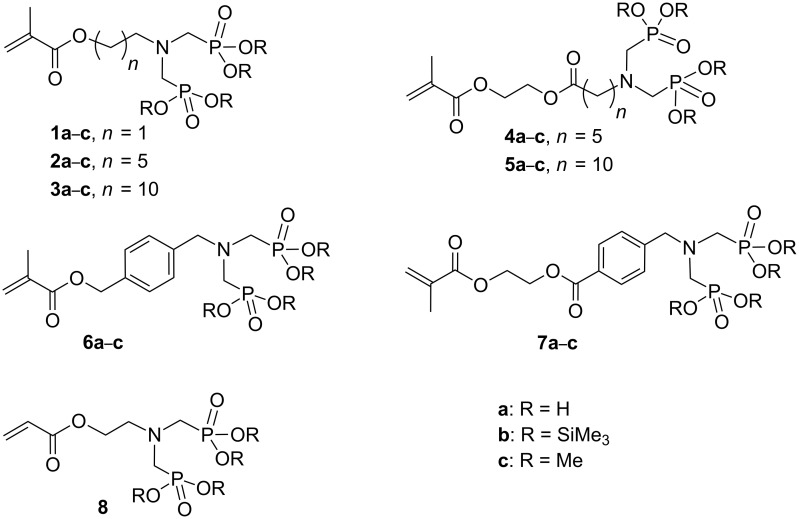
Novel bisphosphonate methacrylate monomers.

We have designed new bifunctional monomers **1a**–**7a** bearing a methacrylate and an amino(bismethylene)bisphosphonate (Scheme 1) linked by an aliphatic or an aromatic spacer [20–21].

**Scheme 2:**
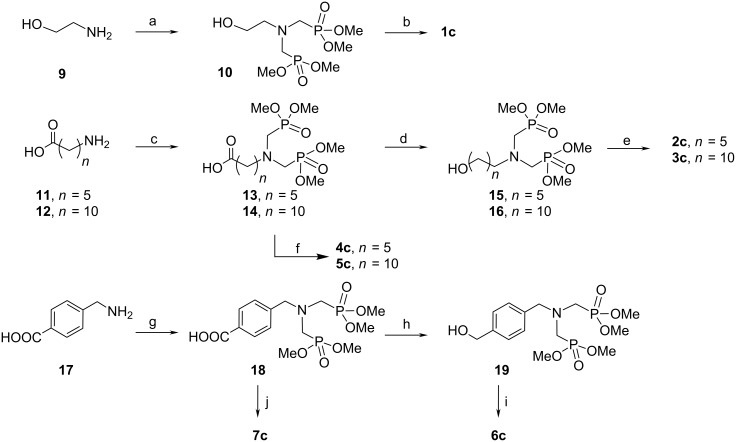
Synthesis of novel bisphosphonate methacrylate monomers **1c**–**7c**, by use of the following reagents and conditions: (a) paraformaldehyde, (MeO)_2_P-OH, THF, reflux, 96%; (b) methacryloyl chloride, NEt_3_, CHCl_3_, 68%; (c) paraformaldehyde, (MeO)_2_P-OH, THF, reflux (*n* = 5, 92%; *n* = 10, 95%); (d) BH_3_-THF, CH_2_Cl_2_ (*n* = 5, 87%; *n* = 10, 85%); (e) methacryloyl chloride, NEt_3_, CHCl_3_ (*n* = 5, 75%; *n* = 10, 77%; (f) HEMA (**22**), DCCI, DMAP, CHCl_3_ (*n* = 5, 74%; *n* = 10, 73%); (g) paraformaldehyde, (MeO)_2_P-OH, THF, reflux, 92%; (h) B_2_H_6_; (i) methacryloyl chloride, NEt_3_, CHCl_3_, 62%; (j) HEMA (**22**), DCCI, DMAP, CHCl_3_, 65%.

To the best of our knowledge, only a single acrylate containing monomer **8** has been previously synthesized and tested, after copolymerization and incorporation, in a desensitizing solution for lithography [22]. More recently we investigated a similar compound **1a** for its adhesive or anticorrosive or flame-retardant properties [20–21]. The synthesis of bisphosphonate monomers **1c**–**7c** is described in Scheme 2.

Thus bisphosphonate **1c** was simply obtained from 2-aminoethanol (**9**) by a two-step process involving first Kabachnik–Fields conditions [23–24] to introduce the bisphosphonate moiety followed by esterification of compound **10** with methacryloyl chloride. The synthesis of the next aliphatic target molecules **2c**–**4c** and **3c**–**5c** started from 6-aminohexanoic acid (**11**) and 11-aminoundecanoic acid (**12**), respectively. The three component coupling of **11**, respectively **12**, with paraformaldehyde and dimethyl phosphite furnished bisphosphonates **13** and **14** in excellent yields. These latter compounds were then reduced regioselectively by diborane [25] to the corresponding alcohols **15** and **16**, respectively. Their subsequent esterification in the presence of methacryloyl chloride gave the target molecules **2c** and **3c**. Alternatively, compounds **13** and **14** were esterified with (hydroxyethyl)methacrylate (HEMA, **22**) to give the monomers **4c** and **5c**. The two aromatic targets **6c** and **7c** were prepared from *p*-(aminomethyl)benzoic acid (**17**) which was converted into the bisphosphonate **18** in 92% yield under Kabachnik–Fields conditions. This common intermediate **18** was either reduced by diborane to the alcohol **19** followed by esterification by methacryloyl chloride giving access to compound **6c**, or esterified directly with HEMA (**22**) to furnish the bisphosphonate **7c**.

### Study of bisphosphonate methacrylate monomer deprotection

As already mentioned in the introduction, the resulting polymers from bisphosphonate methacrylate monomers can be involved in many applications such as dental adhesives, ion-exchange resins and adhesion promotors. However, these polymers must be in the acidic form, i.e., with phosphonic acid groups, to function efficiently [26].

**Scheme 3:**
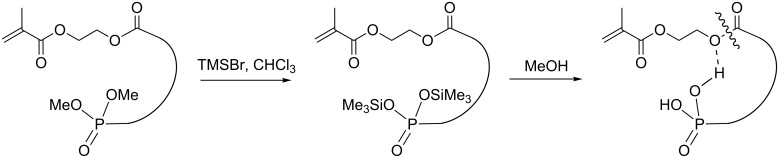
Schematic procedure for phosphonate methacrylate monomer deprotection.

The intermediate bisphosphonates were then subjected to a two-step deprotection process to restore the phosphonic acids by using first trimethylsilyl bromide followed by a methanolysis step [27]. The first step is known to transform alkyl phosphonates into the corresponding trimethylsilyl phosphonates which are then cleaved to the phosphonic acids under hydrolytic conditions [28]. Phosphonates **1c**–**7c** were treated with trimethylsilyl bromide for 16 h at room temperature to give the trimethylsilyl esters **1b**–**7b** which were isolated in quantitative yields (Scheme 3).

^1^H NMR analysis of compounds **1b**–**7b** showed the absence of deprotected products **1a**–**7a** which could have arisen from possible traces of residual HBr. The next challenge was to cleave the trimethylsilyl phosphonates selectively, without affecting alkyl carboxylates, under controlled conditions of both temperature and solvent [23] since alkyl esters including acrylate or methacrylate esters are sensitive to hydrolytic conditions [29]. According to McKenna's recent results, the use of methanol instead of water should achieve the selective deprotection of these trimethylsilyl phosphonates [30]. Our results are summarized in Table 1.

**Table 1:** Deprotection of phosphonates **1b**–**7b** obtained with the following conditions: 1) TMSBr, CHCl_3_, RT, 16 h; 2) MeOH, RT, 2 h.

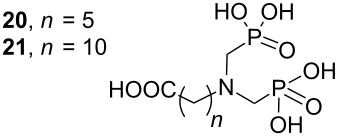			
Entry	Reactant	Product	Yield (%)^a^

1	**1b**	**1a**	95
2	**2b**	**2a**	97
3	**3b**	**3a**	94
4	**4b**	**4a**/**9**/**24**	97 (30/35/35)^b^
5	**5b**	**5a**/**9**/**25**	98 (30/35/35)^b^
6	**6b**	**6a**	98
7	**7b**	**7a**	99

^a^Crude yield after solvent evaporation. ^b^Relative proportions as determined by ^1^H NMR.

The crude silyl esters **1b**–**7b** were dissolved in methanol and stirred for 2 h at ambient temperature. Concentration of the reaction mixture furnished phosphonic acids **1a**–**3a** and **6a**, **7a** in good yields (Table 1, entries 1 and 2) while methanolysis of compounds **4b** and **5b** resulted in a mixture of the desired phosphonic acids **4a** and **5a**, HEMA (**22**) and the phosphonic acids **20** and **21**, respectively (entries 4 and 5). In both the latter cases careful ^1^H NMR examination of the reaction mixture from the methanolysis step revealed that the phosphonic acid **4a**, respectively **5a**, was the sole compound until evaporation of the solvent. These results showed that the internal carboxylic ester was only cleaved after concentration of the reaction mixture probably due to the higher acidity of the medium and are in agreement with the previously described deprotections using water as solvent [31].

We prepared two model compounds **24** and **25** derived from acetylation of HEMA (**22**) and (hydroxybutyl)methacrylic acid (HBMA, **23**), respectively to study the deprotection of these esters in the methanol under increasing concentrations of hydroxymethylphosphonic acid as a model phosphonic acid. Our results are summarized in Table 2.

**Table 2:** Deprotection of esters **24** and **25**^a^.

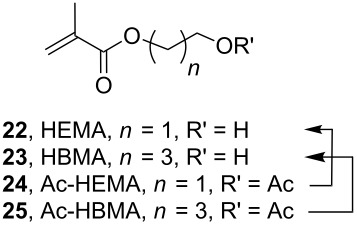			
Entry	Acid amount^b^	Yield of 9^c^	Yield of 10^c^

1	2	30	20
2	4	50	40
3	6	62	54
4	8	70	65
5	10	75	70

^a^Starting esters were stirred in MeOH at RT for 4 h before concentration of the solvent. ^b^Molar percentage of hydroxymethylphosphonic acid. ^c^Isolated yield after evaporation and chromatographic purification.

We observed that: i) Both ester functions were stable until evaporation of the mixture, ii) the sole acetyl group was cleaved by concentrating the reaction mixture leading to increasing proportions of HEMA (**22**), respectively HBMA (**23**) as the molar percentage of hydroxymethylphosphonic acid increases. These last results are in agreement with the observed cleavage of compounds **4a** and **5a** and show the weak influence of the chain length between the methacrylate and acetate groups. It is worth mentioning that the phosphonic acid **7a** is stable in the two-step deprotection process (Table 1, entry 7) emphasizing the greater stability of conjugated carboxylic esters over unconjugated ones.

Other examples of similar phosphonate deprotection by TMSBr involved the presence of a tertiary amine but the authors did not mention any cleavage of the carboxylic ester to prove the role of the base used during the selective deprotection of the phosphonic ester into its acid [32]. We finally deprotected the trimethylsilyl phosphonates **4b** and **5b** with methanol in the presence of aqueous ammonia (reaction time 1 h) to obtain the target phosphonic acids **4a** and **5a** in quantitative yields as their ammonium salts.

## Conclusion

In conclusion we were able to prepare new bifunctional monomers bearing a methacrylate function and a bisphosphonic acid function. We confirmed that the unconjugated alkyl ester function involved in these monomers was cleaved selectively in the presence of conjugated esters by the released phosphonic acid. The use of methanol instead of water during this final deprotection step was essential to preserve the more stable methacrylate and benzoate esters.
